# The impact of HTLV-1 infection on clinical and immunological outcomes in patients coinfected with HIV and hepatitis C virus

**DOI:** 10.1186/1742-4690-8-S1-A54

**Published:** 2011-06-06

**Authors:** Fabianna Bahia, Chloe Le Marchand, Jennifer Evans, Eduardo Netto, Kimberly Page, Carlos Brites

**Affiliations:** 1 Federal University of Bahia, Bahia, Brazil; 2 University of California, San Franscisco, CA, USA

## Introduction

HIV, hepatitis C (HCV), and human T-cell lymphotropic virus I (HTLV-1) are associated with high global burdens of disease, notably in resource-poor locales. They share similar routes of transmission and cause chronic infections with associated morbidity. We performed a cross-sectional study to assess the impact of HTLV-1 infection on clinical outcomes in HIV/HCV co-infected patients.

## Methods

We enrolled 102 (72.3%) with HIV/HCV co-infection (Group 1) and 39 (27.7%) triply infected with HIV, HCV, and HTLV-1 (Group 2). We reviewed medical records of two groups of patients followed in two outpatients services in Salvador, Brazil. We collected and compared demographic, behavioral-related information, immunological, virological and histological parameters for HIV-1 and HCV infection.

## Results

Demographics, virological and immunological characteristics were similar in the two groups; a higher proportion of triply infected patients (Group 2) reported any history of injection drug use (IDU) compared to dually infected (Group 1) patients (75% vs. 45.8%; p =0.003). No differences were seen between groups in HIV clinical outcomes (CD4 count and viral load). Alanine aminotransferase levels were significantly higher in HIV/HCV co-infected patients (p =0.045). Liver fibrosis damage based on Metavir scores were similar between groups (0.97) but were worse with lower CD4 cell count (under 200cells/mm3) (p = 0.01).

## Conclusions

HIV/HTLV-1 and HIV/HCV coinfections may worsen clinical related outcomes, but virological and immunological outcomes were similar in both groups. Hepatic measures were worse in patients with more severe immunosuppression.

